# Supplementary material for: Neutron crystallography reveals mechanisms used by Pseudomonas aeruginosa for host-cell binding
Source: Nat Commun. 2022 Jan 11;13:194. doi: 10.1038/s41467-021-27871-8 (PMC8752737; doi:10.1038/s41467-021-27871-8)
Supplement: Supplementary file 1 — Supplementary information [file 41467_2021_27871_MOESM1_ESM.pdf]

Supplementary Information

Supplementary Table 1: Geometrical parameters of the hydrogen-bonding network in the four binding sites of the LecB/Fuc-d_{12} complex in the neutron structure (with deuterium atom attached to donor with constrained distance of 0.85 Å during refinement procedure)

Donor	Acceptor	Hydrogen bond distance (Å)/ angle (°)			
		Chain A	Chain B	Chain C	Chain D
Direct interactions between the fucose residue and the protein					
**Fuc-O2**	Asp96.OD1	2.59	2.52	2.48	2.57
**Fuc-O3**	Asp99.OD2	2.51	2.53	2.49	2.43
**Fuc-O4**	Gly114.OXT[a]	2.49	2.47	2.48	2.51
**Ser23.N**	Fuc-O5	2.92	2.96	2.94	2.96
**Fuc-OD2**	Asp96.OD1	1.81/165.1	1.74/179.1	1.77/151.5	1.86/162.6
**Fuc-OD3**	Asp99.OD2	1.74/160.0	1.77/161.8	1.92/133.3	1.69/153.7
**Fuc-OD4**	Gly114.OXT[a]	1.75/148.2	1.83/137.3	1.76/153.4	1.77/165.4
**Ser23.ND**	Fuc-O5	2.10/169.8	2.14/169.1	2.12/176.4	2.16/171.2
Water-bridged hydrogen bonds between the fucose residue and the protein					
**Thr98.ND**	O (Wat1)	2.12/162.1	2.05/155.8	2.15/164.1	2.01/164.1
**Fuc-OD1**	O (Wat1)	-	2.48/170.4	-	2.40/162.6
**D1 (Wat1)**	Fuc-O1	2.46/144.4	-	-	-
**D1 (Wat1)**	Fuc-O2	2.43/142.2	-	2.54/143.0	-
**D2 (Wat1)**	Fuc-O1	-	-	2.56/125.6	-
**Fuc-OD1**	O (Wat2)	2.24/160.8	-	-	-
**D2 (Wat2)**	Ser23.OG	1.87/149.5	-	-	-
**D1 (Wat2)**	Fuc-O1	-	-	-	2.50/147.9
**D2 (Wat2)**	Ser23.OG	-	-	-	2.40/160.7

[a] C-terminal residue from the neighbouring monomer

Supplementary Table 2: Geometrical parameters of the hydrogen-bonding network in binding sites of chains A and D with deuterium located in the omit map, with no constraints for O-D distance on fucose hydroxyl groups during refinement procedure. As defined in the results section on the importance of calcium, the hydrogen bonds involving Fuc-O5/Ser23 is a normal hydrogen bond; Fuc-O2/Asp96 and FucO4/Gly114 are short but classical; Fuc-O3/Asp99 is considered a low-barrier hydrogen bond because of the position of the hydrogen equally shared by the two heteroatoms.

			Chain A	Chain D	average	St. Dev
Dist donnor...acceptor (Å)	Fuc-O2	Asp96.OD1	2.59	2.57	2.58	0.01
O-D (Å)	Fuc-O2	D	0.94	0.76	0.85	0.09
D....O (Å)	D	Asp96.OD1	1.68	1.86	1.77	0.09
Angle O-D…O (°)	O2-D...Asp		161.9	153.2	157.6	4.4
Dist donnor...acceptor (Å)	Fuc-O3	Asp99.OD2	2.51	2.43	2.47	0.04
O-D (Å)	Fuc-O3	D	1.5	1.09	1.30	0.21
D....O (Å)	D	Asp99.OD2	1.03	1.38	1.21	0.17
Angle O-D…O (°)	O3-D...Asp		164	160.4	162.2	1.80
Dist donnor...acceptor (Å)	Fuc-O4	Gly114.OXT	2.49	2.51	2.50	0.01
O-D (Å)	Fuc-O4	D	0.71	0.71	0.71	0.00
D....O (Å)	D	Gly114.OXT	2.2	1.91	2.06	0.15
Angle O-D…O (°)	O4-D...Gly		106.3	144.2	125.3	18.9
Dist donnor...acceptor (Å)	Ser23.N	Fuc-O5	2.92	2.96	2.94	0.02
O-D (Å)	Ser23.N	D	1.06	0.81	0.94	0.13
D....O (Å)	D	Fuc-O5	2.35	2.52	2.44	0.09
Angle O-D…O (°)	Ser-D...O5		110.5	115.4	113.0	2.45

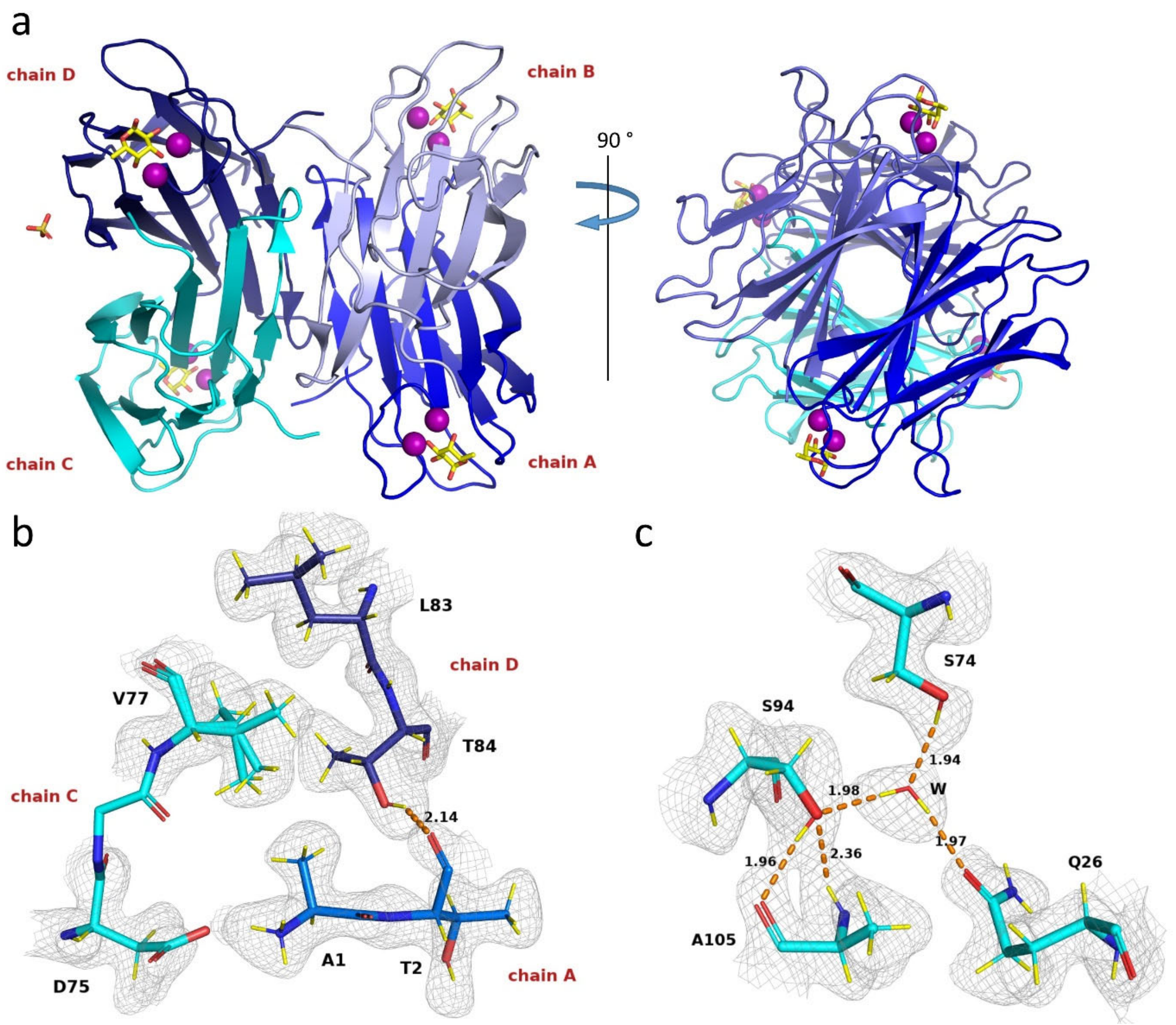

Supplementary Figure 1: Room temperature structure of D-LecB in complex with Fuc-d_{12}. Calcium ions are shown as purple spheres, fucose molecules are shown as yellow sticks, a sulphate ion is shown as orange and red sticks. Hydrogen bonds (Å) are shown as orange dashed lines. Deuterium atoms are coloured yellow (panels b and c). The 2mF_o-DF_c neutron density (grey mesh) is contoured at 0.8σ. (a) Overall structure of D-LecB tetramer in complex with Fuc-d_{12}. (b) Protein interface between chains A, C and D. (c) Hydrogen bonding network involving a water molecule.

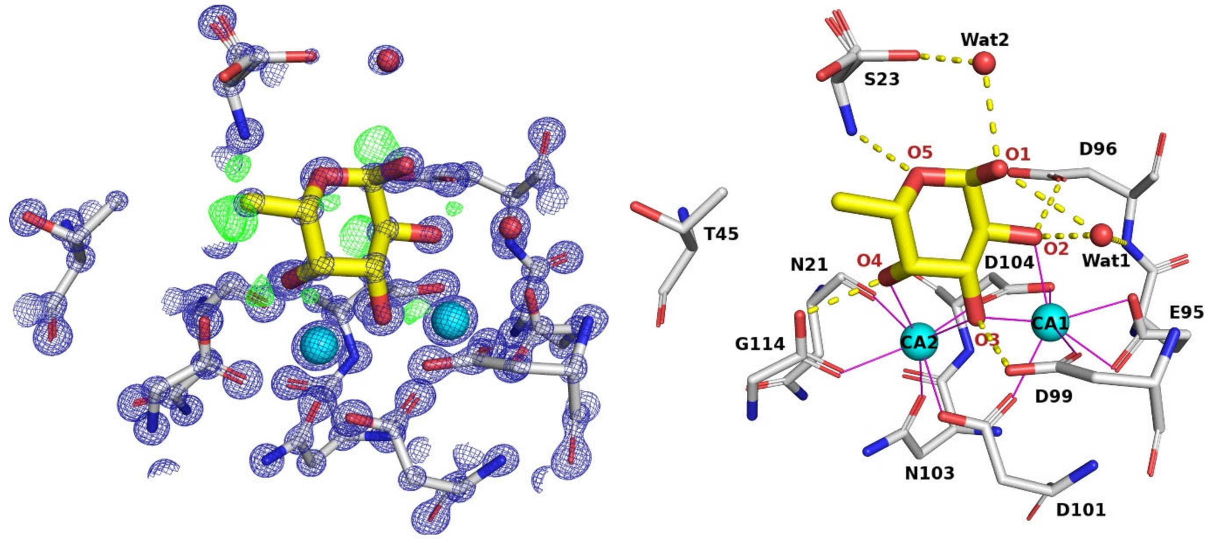

Supplementary Figure 2: Stick representation of the 0.9 Å 100K X-ray structure of the fucose-binding site of the perdeuterated LecB/Fuc-d$_{12}$ complex. Fucose is shown as thick yellow sticks and the protein as thin grey sticks. 2mF_o-DF_c electron density (blue mesh) is contoured at 4σ. The mF_o-DF_c omit electron density (green mesh) is contoured at 2.5σ showing the positions of deuterium atoms on the fucose. Hydrogen bonds are shown as yellow dashed lines and distances are in Å. The metal coordination is represented by purple solid lines. The calcium ions are shown as cyan spheres.

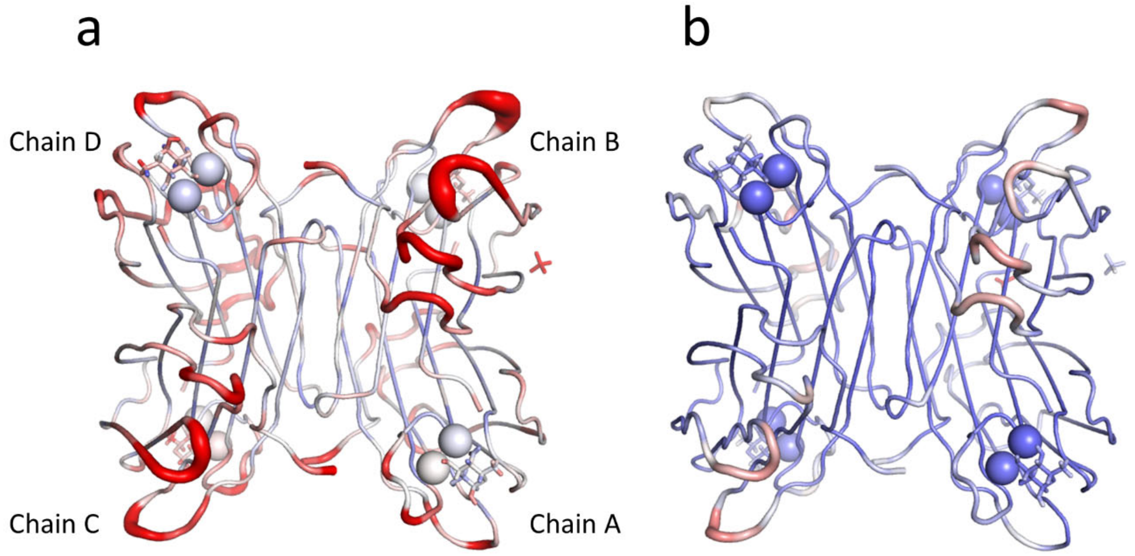

Supplementary Figure 3: Comparison of B-factors at (a) room temperature (neutron/X-ray) and (b) 100K structures (X-ray) of perdeuterated LecB/Fuc-d$_{12}$ complex. Graphical representation was performed with the "cartoon putty" option in Pymol with colour coding from 3 (blue) to 20 (red) for B-factor. Chains A and D display lower B-factors and therefore better quality maps.

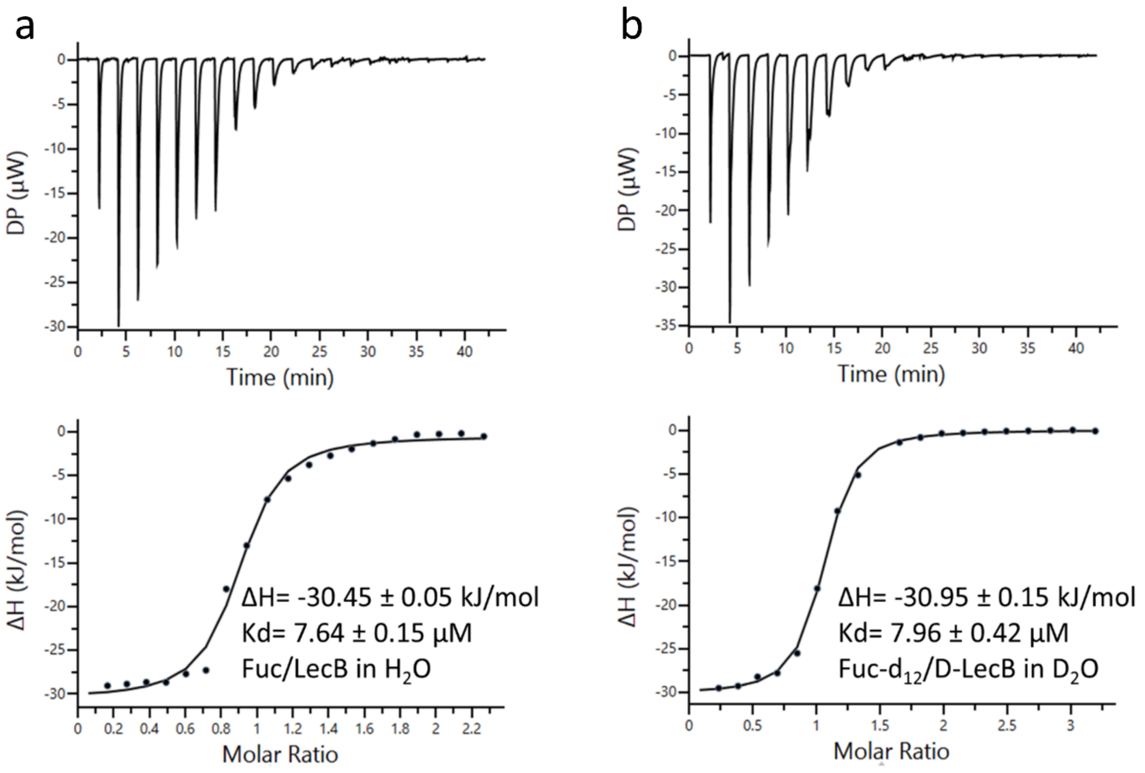

Supplementary Figure 4: Isothermal titration calorimetry results of binding of (a) LecB/L-Fuc in H_2O and (b) D-LecB/L-Fuc-d_{12} in D_2O. Top: data obtained from 20 automatic injections, 2 µL each, of L-fuc (4 mM)/L-Fuc-d_{12} (7.6 mM) into the cell containing LecB (0.46 mM)/D-LecB (0.50 mM). Lower: plot of the total heat released as a function of total ligand concentration for the titration shown above. The solid line represents the best least-squares fit for the obtained data.

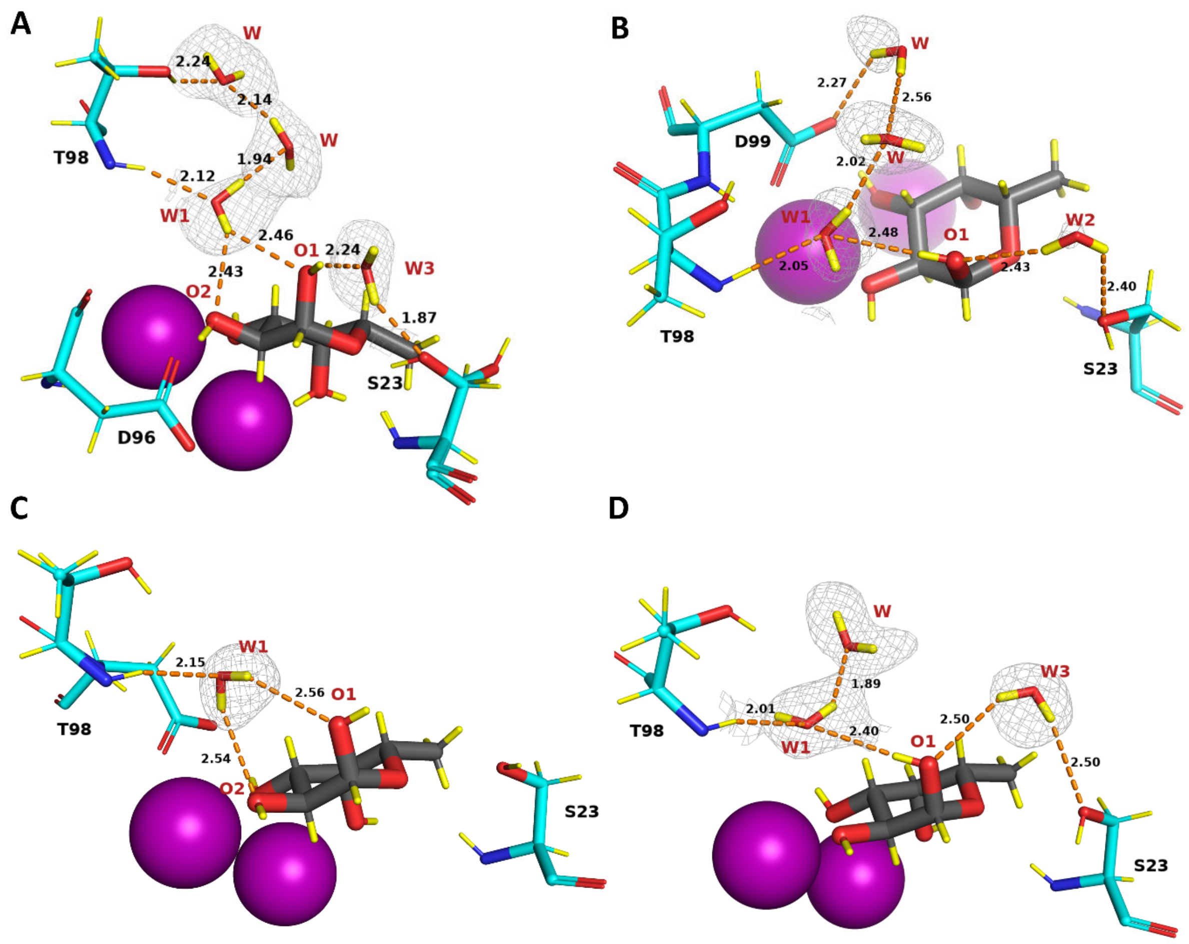

Supplementary Figure 5: Stick representation of the fucose-binding sites in chains A, B, C and D of the perdeuterated LecB/fucose tetrameric complex. The 2mF_o-DF_c neutron density map (grey mesh) is contoured at 0.7σ and is showing highly ordered and contacting water molecules in the binding sites. Hydrogen bonds are shown as orange dashed lines with distances in Å. Fucose is depicted as grey sticks. Deuterium atoms are coloured yellow. Calcium ions are shown as purple spheres.

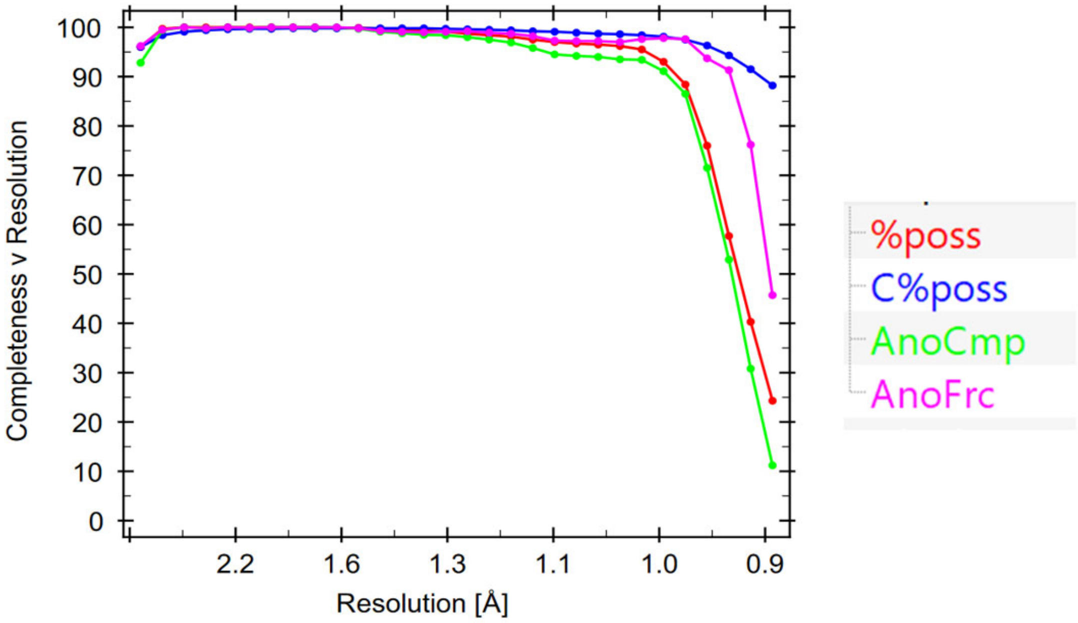

Supplementary Figure 6: Completeness vs. resolution curve for the 0.9 Å X-ray structure of the perdeuterated LecB/Fuc-d$_{12}$ complex collected at 100K. %poss is completeness in the shell. C%poss is the cumulative completeness to that resolution. AnoCmp are the anomalous completeness values that are the percentage of possible anomalous differences measured. AnoFrc is the % of measured acentric reflections for which an anomalous difference has been measured.
