# Supplementary material for: Neutron crystallography reveals mechanisms used by Pseudomonas aeruginosa for host-cell binding
Source: Nat Commun. 2022 Jan 11;13:194. doi: 10.1038/s41467-021-27871-8 (PMC8752737; doi:10.1038/s41467-021-27871-8)
Supplement: Supplementary file 3 — Reporting Summary [file 41467_2021_27871_MOESM3_ESM.pdf]

nature portfolio

Corresponding author(s): Anne Imberty

Last updated by author(s): Nov 25, 2021

Reporting Summary

Nature Portfolio wishes to improve the reproducibility of the work that we publish. This form provides structure for consistency and transparency in reporting. For further information on Nature Portfolio policies, see our Editorial Policies and the Editorial Policy Checklist.

Statistics

For all statistical analyses, confirm that the following items are present in the figure legend, table legend, main text, or Methods section.

n/a	Confirmed	
☐	☒	The exact sample size (*n*) for each experimental group/condition, given as a discrete number and unit of measurement
☐	☒	A statement on whether measurements were taken from distinct samples or whether the same sample was measured repeatedly
☒	☐	The statistical test(s) used AND whether they are one- or two-sided<br>*Only common tests should be described solely by name; describe more complex techniques in the Methods section.*
☒	☐	A description of all covariates tested
☒	☐	A description of any assumptions or corrections, such as tests of normality and adjustment for multiple comparisons
☐	☒	A full description of the statistical parameters including central tendency (e.g. means) or other basic estimates (e.g. regression coefficient) AND variation (e.g. standard deviation) or associated estimates of uncertainty (e.g. confidence intervals)
☒	☐	For null hypothesis testing, the test statistic (e.g. *F*, *t*, *r*) with confidence intervals, effect sizes, degrees of freedom and *P* value noted<br>*Give P values as exact values whenever suitable.*
☒	☐	For Bayesian analysis, information on the choice of priors and Markov chain Monte Carlo settings
☒	☐	For hierarchical and complex designs, identification of the appropriate level for tests and full reporting of outcomes
☒	☐	Estimates of effect sizes (e.g. Cohen's *d*, Pearson's *r*), indicating how they were calculated

Our web collection on statistics for biologists contains articles on many of the points above.

Software and code

Policy information about availability of computer code

Data collection	Data collection software available on LADI-III instrument at the ILL was used for the neutron data collection. The Mardtb345 software was used to collect the room-temperature X-ray data set on the GeniX 3D Cu High Flux diffractometer at the IBS. The MXCuBE2 software was used to collect the 100K X-ray structure on Proxima-1 beamline at the SOLEIL synchrotron. ITC data were collected using MicroCal Origin software (v.7.0).
Data analysis	Following programs and softwares were used in this study: LAUEGEN (v6.0), LSCALE, Scala (v.3.3.16), CCP4 suite (v7.1), Phenix (v1.16), iMosflm (v7.3.0), Coot (v0.8.9.2), Pymol (v2.3.2), MicroCal PEAQ-ITC Analysis Software (v1.0.0.1259), MolProbity (v.4.5).

For manuscripts utilizing custom algorithms or software that are central to the research but not yet described in published literature, software must be made available to editors and reviewers. We strongly encourage code deposition in a community repository (e.g. GitHub). See the Nature Portfolio guidelines for submitting code & software for further information.

Data

Policy information about availability of data

All manuscripts must include a data availability statement. This statement should provide the following information, where applicable:

- Accession codes, unique identifiers, or web links for publicly available datasets
- A description of any restrictions on data availability
- For clinical datasets or third party data, please ensure that the statement adheres to our policy

Coordinates and structure factors have been deposited in the Protein Data Bank under the accession codes: 7PRG for the room temperature X-ray/neutron structure of the perdeuterated LecB/fucose complex and 7PSY for the 100K X-ray structure of perdeuterated complex. All other structures of LecB lectin used for comparisons were taken from the Protein Data Bank under accession codes: 1GZT, 1OXC, 1UZV.

Field-specific reporting

Please select the one below that is the best fit for your research. If you are not sure, read the appropriate sections before making your selection.

- [x] Life sciences
- [ ] Behavioural & social sciences
- [ ] Ecological, evolutionary & environmental sciences

For a reference copy of the document with all sections, see nature.com/documents/nr-reporting-summary-flat.pdf

Life sciences study design

All studies must disclose on these points even when the disclosure is negative.

Sample size	No sample size was determined for this study since it was not necessary for the structural analysis. The protein of interest was crystallized and several data sets were collected in this study. Two room-temperature data sets (X-ray and neutron) and one 100K X-ray data set were collected from different crystals of the same protein sample.
Data exclusions	No data were excluded in this study.
Replication	ITC measurements were done in at least two replicates. Protein crystallization trials were replicated using several batches of the purified protein. All attempts were successful.
Randomization	Randomization was applied during structure refinement where a random subset of test reflections (5 %) was used for cross-validation. No other randomization was applied in this study.
Blinding	Blinding was not applicable in this study since there was no group allocation.

Reporting for specific materials, systems and methods

We require information from authors about some types of materials, experimental systems and methods used in many studies. Here, indicate whether each material, system or method listed is relevant to your study. If you are not sure if a list item applies to your research, read the appropriate section before selecting a response.

Materials & experimental systems

n/a	Involved in the study	
x		Antibodies
x		Eukaryotic cell lines
x		Palaeontology and archaeology
x		Animals and other organisms
x		Human research participants
x		Clinical data
x		Dual use research of concern

Methods

n/a	Involved in the study	
x		ChIP-seq
x		Flow cytometry
x		MRI-based neuroimaging
